# Parental smoke exposure and the development of nicotine craving in adolescent novice smokers: the roles of *DRD2*, *DRD4*, and *OPRM1* genotypes

**DOI:** 10.1186/s12890-015-0114-z

**Published:** 2015-10-08

**Authors:** Marloes Kleinjan, Rutger C.M.E. Engels, Joseph R. DiFranza

**Affiliations:** Trimbos Institute (Netherlands Institute of Mental Health and Addiction), Da Costakade 45, 3521 VS Utrecht, The Netherlands; Behavioural Science Institute, Radboud University Nijmegen, Montessorilaan 3, 6525 HR Nijmegen, The Netherlands; University of Massachusetts Medical School, 55 N Lake Ave, Worcester, MA 01655 USA

**Keywords:** Craving, Early adolescents, Novice smokers, Genes, Parental smoke exposure

## Abstract

**Background:**

Among adolescent novice smokers, craving is often the first, and is the most reported, symptom of nicotine dependence. Until now, little has been known about the development of craving symptoms in novice smokers. The aim of this study was to identify specific genetic (i.e., *DRD2* Taq1A, *DRD4* 48 bp VNTR, and *OPRM1* A118G polymorphisms) and environmental mechanisms that underlie the emergence of both cue-induced and cognitive craving among adolescent novice smokers.

**Method:**

A five-wave longitudinal, genetically-informed survey study was conducted with intervals of four months. The sample included 376 early adolescent smokers (12–13 years of age at baseline). Self-report questionnaires were completed regarding smoking behavior, observed parental smoking behavior, and both cue-induced and cognitive craving.

**Results:**

Data were analyzed with a latent growth curve approach. For both cue-induced and cognitive craving, significant interaction effects were found for *DRD2* Taq1A with parental smoke exposure. A1-allele carriers did not seem to be influenced by the environment with regard to craving development. Adolescents who are homozygous for the A2-allele and who are more exposed to parental smoking experience the highest levels of both types of craving over time. No significant interaction effects were found between parental smoke exposure and *DRD4* 48 bp VNTR or *OPRM1* A118G.

**Conclusions:**

Previous studies identified *DRD2* Taq1A A1-allele carriers as vulnerable to developing nicotine dependence. However, this study showed that parental smoking increased the chances of developing dependence more rapidly for early adolescents who are considered to be *less* sensitive to the rewarding effects of nicotine according to their DRD2 Taq1A genotype. It is thus especially important that these young people not be exposed to smoking in their social environment.

**Electronic supplementary material:**

The online version of this article (doi:10.1186/s12890-015-0114-z) contains supplementary material, which is available to authorized users.

## Background

Tobacco has been found to be one of the most addictive recreational substances, and a significant proportion of those experimenting with smoking progress to dependence [1]. The literature provides strong support for the presence of withdrawal symptoms among light and intermittent smokers [2–4]. Craving, which refers to the desire or urge to smoke, is commonly the first symptom of dependence and can occur within days of initiating smoking [2]. Among adolescent smokers, craving is by far the most reported dependence symptom; moreover, compared to other withdrawal symptoms, experienced craving forms the single most important barrier to both the attempt to quit and successful cessation of smoking [5, 6].

The Sensitization-Homeostasis Model (SHM) offers a theoretical framework for understanding the development of nicotine dependence [7]. The SHM implies that neurophysiological processes underlying nicotine dependence can be set in motion when smoking one’s first cigarette. More specifically, the SHM proposes two distinct processes that induce craving during the phases of initiation and intermittent smoking. First, the very first dose of nicotine is argued to set off a cascade of neurological events in the brain involving, amongst others, dopamine. When one smokes a cigarette, nicotine causes the release of dopamine from the mesolimbic dopamine system, and the release of dopamine consequently inhibits craving. The craving symptoms that initially occur can be controlled by smoking approximately one cigarette per week. However, as tolerance increases, the duration of relief offered by each cigarette shortens progressively [7]. If the smoker does not restrain consumption, the latency to craving shortens progressively over time, and this latency is the primary determinant of changes in smoking frequency [8]. In addition to being a symptom of nicotine withdrawal, craving can also be evoked in the absence of withdrawal by cues that have been previously paired with nicotine intake (e.g., sensory or situational cues, such as the smell of cigarette smoke).

Consistent with the SHM, studies among adults have shown that differences in craving levels could be partly explained by genetic variation in the dopamine pathway – that is, by variations in polymorphisms in the D2 and D4 dopamine receptor gene (*DRD2* Taq1A (rs1800497) and *DRD4* 48 bp VNTR) [9–11]. Activation along mesolimbic dopamine substrates thus seems to influence the motivational and appetitive properties (i.e., craving) of tobacco. The *DRD2* Taq1A and *DRD4* 48 bp VNTR polymorphisms are associated with differences in dopamine binding potential and could therefore potentially play a role in the variation in experiencing reward after nicotine intake [12]. Considering that craving can occur within days after initiation, *DRD2* Taq1A and *DRD4* 48 bp VNTR may partly shape the development of craving in novice smokers.

Studies on genetic variation in adolescent craving are scarce. One study showed that *DRD2* Taq1A seems to be associated with both smoking continuation and progression to nicotine dependence in adolescents [13]. *DRD4* 48 bp VNTR has been associated with smoking more frequently (implying a shorter latency to craving) [14, 15] but not directly with measures of nicotine dependence [13]. Also, a recent review concluded that there was no strong evidence supporting an association between the *DRD2* Taq1A polymorphism and smoking behavior [16]. So far, no studies have looked at the association between the *DRD2* Taq1A and *DRD4* 48 bp VNTR polymorphisms in the initial occurrence and progression of craving in early phases of tobacco use. Narrowly defined phenotypes such as the initial development of craving might be better suited for molecular genetic studies than the often-used Fagerström Test for Nicotine Dependence [17] or DSM-categorizations for nicotine dependence (DSM-III-R, DSM-IV or DSM-V; American Psychiatric Association, 1987, 1994, 2013), which are not principally designed to measure the earlier stages of nicotine dependence.

In addition to genetic polymorphisms in candidate genes related to dopaminergic neurotransmission, genetic polymorphism related to the opioid system may also affect the onset and progression of craving symptoms. According to the incentive salience theory, the dopaminergic processes may become particularly relevant after repeated exposure to nicotine [18]. The initial phase of nicotine intake is thought to be mainly characterized by opioid neurotransmission, which is involved in the subjective pleasure (‘liking’) derived from the substance [19]. ‘Liking’ processes and opioid neurotransmission may thus be particularly relevant in the initiation phase. We will therefore also include the μ-opioid receptor *OPRM1* A118G polymorphism (rs1799971). A recent review on the role of *OPRM1* A118G in smoking behaviour suggests that there is evidence for the involvement of the *OPRM1* 118A-allele in nicotine reinforcement and dependence [20]. Only one of the reviewed studies looked at craving as an aspect of nicotine dependence specifically, and it found no relation between *OPRM1* A118G and craving [21]. Also, studies on the *OPRM1* A118G genotype in adolescent samples are scarce. To our knowledge, only one study investigated the *OPRM1* A118G genotype in relation to the development of adolescent smoking; that study found that males who are homozygous for the 118A-allele showed faster development in smoking frequency (implying a role of the 118A-allele in shortening the latency to craving), whereas in females the 118G-allele was associated with faster development of smoking frequency [22].

The second process described by the SHM holds that smoking-related environmental cues can stimulate craving. The release of dopamine functions as a signal that a rewarding stimulus has been encountered and initializes the association of tobacco-related environmental stimuli with the effects of nicotine. Sensory stimuli associated with the act of smoking, such as the smell of smoke, can become cues. Several laboratory studies have shown that exposure to smoking-related cues is associated with robust increases in self-reported craving, modest physiological responses, biases in attentional processing of smoking-related cues, shorter latency to smoking and increased smoking behavior [23–26]. Since nicotine dependence can develop quickly among novice smokers [7], and since the regulatory executive system is not yet fully developed in adolescents [27, 28], adolescent smokers in the early stages of smoking may encounter challenges in regulating or inhibiting cue-induced appetitive response tendencies that result from exposure to environmental smoking cues. A greater exposure to smoking cues in adolescent novice smokers may thus contribute to a shortening of the latency to craving and to the subsequent choice to smoke a cigarette. In early adolescence, parental smoking is one factor that seems to be associated with the risk for nicotine dependence [29, 30]. The association between parental smoking and the risk of craving and subsequent dependence may be explained by physiological as well as psychosocial processes. Previous research has shown that exposure to high levels of tobacco smoke in the social environment is capable of producing plasma nicotine concentrations that are comparable to concentrations found in active smokers [31–33] and exposure can engender substantial brain nicotinic acetylcholine receptor occupancy [34]. In theory, frequent or prolonged exposure to nicotine absorbed from passive smoke may predispose nicotine-naive adolescents to develop nicotine dependence once they initiate smoking. Animal studies have provided preliminary evidence of physiological processes resulting from passive smoking exposure [35, 36]. On the other hand, psychosocial processes may also explain the association between parental smoking and an increased risk of nicotine dependence in youth. Parental smoking may shape cognitive responses to tobacco use (e.g., through outcome expectancies) as well as actual smoking behaviour (e.g., through social modelling or social norms), which may enhance adolescents’ risk of developing nicotine dependence. Associations between environmental smoking and social-cognitive variables such as smoking outcome expectancies and normative perceptions have been established by a variety of studies [37–39]. Even though the exact mechanisms of action need to be further elucidated, it is well-established that parental smoking affects adolescent initiation of smoking, as well as adolescents’ smoking frequency and progression towards nicotine dependence [29, 40–42]. The level of parental smoking exposure is therefore expected to influence the initial development of craving symptoms in early adolescent novice smokers.

According to the SHM, both genetic and environmental factors could be involved in shaping the development of craving symptoms in early adolescent novice smokers. For some time now, it has been acknowledged that it is not sufficient to know to what extent genes or environment affect behavior; rather, the interplay between the two should be studied to better understand individual differences in certain phenotypes, such as symptoms of nicotine dependence. More specifically, certain genetic polymorphisms could increase the likelihood that adolescents will develop nicotine dependence, but the actual manifestation of nicotine addiction might depend on environmental factors, such as parental smoking exposure [43, 44]. So far, candidate gene studies examining gene-environment effects on smoking and nicotine dependence are scarce. One such study recently examined interactions between polymorphisms of the dopaminergic system (i.e., *DRD2* Taq1A, *DRD4* 48 bp VNTR and *DAT1*) and smoking behavior of parents, siblings and friends in predicting smoking initiation [45]. No significant interaction effects were reported. However, behavioral genetic studies showed that shared environmental factors played a major role in smoking initiation, whereas the influence of genetic factors increased in smoking persistence [46, 47]. In this regard, a recent study found an interaction effect of the serotonin transporter genotype *5-HTTLPR* and poor family environment on smoking habits as well as nicotine and cotinine levels [48]. Based on the notions of the SHM, dopaminergic involvement is likely in the development of craving symptoms; thus, we will test whether dopamine-related genotypes interact with parental smoking exposure in predicting increases in craving over time in novice smokers. We will focus on two different types of craving based on the classification scheme proposed by Singleton and Gorelick [49]. This scheme comprises two general categories of craving models: (1) models based on conditioned mechanisms and (2) models based on cognitive mechanisms. Conditioning or cue-reactivity models posit that nicotine-related cues trigger craving through conditioned neurologic responses. Conversely, cognitive models are based on the assumption that responses to nicotine and nicotine-related cues involve various cognitive processes, such as expectations regarding the pleasant effects of nicotine. Based on the literature described above, it is expected that the development of both cue-induced and cognitive craving over time will be predicted by an interaction between parental smoking exposure and the *DRD2* Taq1A, *DRD4* 48 bp VNTR and *OPRM1* A118G genotype. Identifying specific genetic and environmental mechanisms underlying the emergence of craving among adolescent novice smokers can be expected to lead to a more refined understanding of the etiology of nicotine dependence and to provide important and useful information about the individual susceptibility of adolescents to developing nicotine dependence.

## Method

### Procedure and participants

A total of 1,399 adolescents (52.6 % female) with an average age of 12.63 years (range: 11 – 14, *SD* = 0.59) were recruited from 22 schools in the Netherlands at Time 1 (T1). Across the five waves, 1,360 (97.2 %), 1,230 (87.9 %), 1,183 (84.6 %), 1,188 (84.9 %), and 1,099 (78.1 %) adolescents participated at Time 1 (T1), Time 2 (T2), Time 3 (T3), Time 4 (T4), and Time 5 (T5), respectively. The time between each of the five waves was approximately four months. Adolescents were considered smokers if they reported having tried smoking at least once during one or more of the five measurement waves. This inclusion criterion was chosen because of the notion that one’s very first cigarette may already initiate a series of neurological changes in the brain [7].

A total of 476 of the 1,399 respondents (34.8 %) indicated that they had smoked at the time of one or more of the five waves. Adolescents who indicated that they had smoked were referred to the questions concerning craving. Of the 476 smokers, we included only those smokers who completed at least three waves and were genotyped (*N* = 396).

Of the 396 participants who fulfilled these criteria, 53.3 % were male, and the average age was 12.63 (SD = 0.59) (also see Table 1). Most of the adolescents were of Dutch origin. The participants were also taking part in a larger longitudinal study that started in January 2010, focusing on genetic and environmental influences on substance use among Dutch adolescents. The participants were in the first grade of secondary school at T1. At T1, saliva samples were collected for DNA extraction (Oragene, DNA Genotek Inc). Active, written informed consent for gene analysis was obtained from the adolescents as well as their parents. During each wave, the participants filled out an online or paper-and-pencil questionnaire during school hours. The principal investigator visited every school, personally provided information about the study to teachers and students and was present during the first assessment. Students were explicitly told that all questions were about their regular patterns and not exceptional situations (e.g., holidays), unless otherwise stated. The research design for this study was evaluated and ethically approved by an independent medical ethical committee (METiGG, Utrecht, The Netherlands).

**Table 1:** Descriptive statistics for control and independent model variables

Variable	Response categories	Mean (SD)	Response frequencies	Skewness (SD)
Sex	Male		53.3 %	.13
	Female		46.7 %	(.12)
Education	University preparatory		8.9 %	-.19
	Senior general		22.0 %	(.12)
	Junior general		34.2 %	
	Preparatory vocational		34.8 %	
Ethnicity	Not Dutch		4 %	-.47
	Dutch		96 %	(.12)
Age of smoking initiation		11.67 (1.90)		
Baseline smoking	<1 per day		83.7 %	2.70
	1-5 per day		9.6 %	(.21)
	6-10 per day		5.2 %	
	>10 per day		1.4 %	
DRD2 Taq1A	A2A2		64.2 %	.59
	A2A1/A1A1		35.8 %	(.12)
DRD4 48 bp	<7-repeat		64.2 %	.60
VNTR	≥7-repeat		35.8 %	(.12)
OPRM1 A118G	AA		78.7 %	1.41
	AG/GG		21.3 %	(.12)
Parental smoke exposure	No smoking parents		54.0 %	1.00
	Smoking parent(s)/no exposure		10.2 %	(.13)
	One smoking parent/exposed		22.5 %	
	Two smoking parents/exposed by one		2.3 %	
	Two smoking parents/exposed by both		11.0 %	

### Power analysis

We conducted power analyses using the software Quanto [50]. For the power calculation, we applied the gene-environment interaction design option for continuous outcomes with independent individuals. We used a previous nation-wide study on smoking behaviour among Dutch adolescents aged 15–16 to determine the input for our power analyses [51, 52]. In this study, craving was measured by four items on a six-point scale. Craving mean scores on this measure were around 2.3 (SD = 1.03). Since our target group is smokers aged 12–13, we take a more conservative approach with anticipated mean craving scores of 1.5 (SD = 0.75) in our own sample. The R^2^ for the environmental effect was fixed at 0.035, and the prevalence of smoking in the environment was fixed at 25 % (Environment; Population Prevalence: 0.25) [53]. Main effects of genetic variation are generally quite low; thus, the R2 for the genetic effect was fixed at 0.001. Further, approximately 30 % of the sample was expected to have the *DRD2* Taq1A A1-allele [13], and a dominant model for the A1-allele is assumed [13, 54]. To detect a small interaction effect with an R^2^ of 0.02 with 80 % power (alpha = .05), the sample size required to detect an interaction effect is 374 individuals. Hence, with a sample size of 396 adolescents, we should be able to detect even small interaction effects with *DRD2* Taq1A. Following the same procedure for *DRD4* 48 bp VNTR and *OPRM1* A118G, with 30 % and 20 % of the sample expected to have the risk-alleles (>7 repeats for *DRD4* and the presence of the G-allele for *OPRM1* A118G), 374 was indicated to be sufficient to detect a significant interaction effect.

### Genotyping

DNA was isolated from saliva using the Oragene system (DNA Genotek Inc., Kanata, Ontario, Canada). The *DRD2* Taq1A (rs1800497) and *OPRM1* A118G (rs1799971) polymorphisms were genotyped using Taqman analysis. The *DRD4* 48 bp repeat polymorphism in exon 3 of the dopamine receptor gene was genotyped using simple sequence length analysis. A detailed description of this procedure is published elsewhere [22, 45]. All genotyping assays have been previously validated, and 5 % duplicates and blanks were included as quality controls during genotyping. Genotyping was performed in a CCKL-accredited laboratory at the Department of Human Genetics of the Radboud University Medical Centre in Nijmegen. Distribution of the *DRD2* Taq1A, the *DRD4* 48 bp VNTR, and the *OPRM1* A118G genotype in the study was in accordance with the Hardy-Weinberg equilibrium (p = .71, p = .53, p = .62, respectively). To maximize the power of the analyses and conform to previous studies that tested *DRD2* Taq1A [55, 70], *DRD4* 48 bp VNTR [10, 56], and *OPRM1* A118G [20–22] in relation to substance use outcomes, we dummy coded the polymorphisms. *DRD2* Taq1A was dummy coded into 1 = non-risk (A2A2) and 2 = risk (A1A2 and A1A1). Participants’ *DRD4* 48 bp VNTR genotype was dummy-coded into two categories: 1 = non-risk (short allele, fewer than 7 repeats) and 2 = risk (7 or more repeat allele carriers, at least one long allele). *OPRM1* A118G genotype was dummy coded into 1 (AA) and 2 (AG and GG).

### Questionnaires

#### Adolescent smoking

Smoking behavior of adolescents was assessed at each wave. Adolescents were asked to report, on a nine-point scale, which stage of smoking applied to them. Response categories ranged from 1 = ‘I have never smoked, not even one puff’ to 9 = ‘I smoke at least once a day’ [29, 42, 57–59] (Please see Additional file 1 for the Questionnaires used).

#### Parental smoking exposure

Parental smoking was assessed using the questions: “Does your father smoke?” and “does your mother smoke?”. Responses options were “no, he/she has never smoked”, “no, he/she quit smoking”, and “yes, he/she smokes”. Responses were recoded into paternal smoking (no, yes), maternal smoking (no, yes), and number of smoking parents (0, 1, 2). Exposure to smoking by father/mother was assessed using the question “Does your father/mother smoke when you are with him/her?” Response options were “yes” and “no.” The final measure on smoking exposure was constructed to reflect (0) no smoking parents, (1) smoking parent(s)/no exposure, (2) one smoking parent/exposed by that parent, (3) two smoking parents/exposed by one parent, and (4) two smoking parents/exposed by both parents. Parental smoking behaviour was highly stable over the five waves, with Spearman correlations ranging between .71 and .89.

#### Cue-induced craving

To assess cue-induced craving, we used the cue-induced craving subscale of the Autonomy Over Tobacco Scale (AUTOS) [60, 61]. The four items are: “When I see other people smoking, I want a cigarette”, “When I smell cigarette smoke, I want a cigarette”, “When I feel stressed, I want a cigarette”, and “After eating, I want a cigarette”. Adolescents were asked to indicate which response option, ranging from 1 (never) to 5 (very often) best described them. In smokers, the AUTOS correlated with cigarette consumption and other measures of tobacco use [60]. Cronbach’s alphas for this scale were .88 (T1), .86 (T2), .89 (T3), .93 (T4), and .90 (T5). An average score for cue-induced craving was computed at each wave. Adolescents who started smoking after T1 were given the lowest mean scale scores for the time points in which they were not smoking yet, indicating no experienced craving (total mean craving score = 1).

#### Cognitive craving

Cognitive craving for tobacco was assessed with five items related to the frequency of missing, desiring, thinking of, or longing for a cigarette; The five items were “I desire to smoke a cigarette,” “I miss a cigarette,” “I look forward to lighting a cigarette,” “I desire to inhale the smoke from a cigarette,” and “I think about the nice feeling of deeply inhaling the smoke from a cigarette” [62]. Answers were on a five-point scale, ranging from ‘never’ to ‘very often’. Cronbach’s alphas for this scale were .95 (T1), .95 (T2), .96 (T3), .95 (T4), and .95 (T5). An average score for cognitive craving was computed at each wave.

### Strategy of analyses

Pearson and Spearman correlations were computed to assess the associations between the model variables. Descriptive statistics will be provided. The relation between the independent variables and the development of craving was examined with a latent growth curve model (LGC) using Mplus [63]. This approach is suitable, as individuals do not start at the same level of craving or progress at the same rate. LGC can determine which variables account for these individual variations.

First, we assessed the single growth curves of cue-induced craving and cognitive craving from Time 1 to Time 5 by estimating the initial level (intercept) and the rate of change over time (slope) for both craving outcome measures. Second, *DRD2* Taq1A, *DRD4* 48 bp VNTR, *OPRM1* A118G, and smoking exposure of parents were included as predictor variables to assess whether these variables were predictive of initial values or growth over time of the three craving measures. In a final step, we examined interaction effects on the initial values and growth between *DRD2* Taq1A and smoking exposure of parents. This process was repeated for the *OPRM1* A118G and *DRD4* 48 bp VNTR genotypes, respectively. All models were controlled for the following variables: sex, age of smoking initiation, educational level, ethnicity and smoking behavior as reported on the first measurement [42]. Within Mplus, parameters were estimated with maximum likelihood estimation with robust standard errors (MLR) to accommodate for skewness of the data. Full information maximum likelihood (FIML) estimation was applied to make use of all available data. The model fit was investigated by the following global fit indices: Chi-square, CFI (good fit when higher than .90), and RMSEA (good fit when lower than .08) [64]. To avoid chance capitalization because of the multiple comparisons, we corrected the alpha, which was considered significant when below 0.01.

## Results

### Attrition analyses

Attrition analyses were conducted in order to examine whether those adolescents who were still participants in the fifth wave of the study (*n* = 1,099; 78.1 %) differed from those who dropped out before the fifth wave (*n* = 300; 22.9 %). *T*-tests and Chi-square tests showed no significant differences (*p* > .05) in age, ethnicity, *DRD2* Taq1A, *DRD4* 48 bp VNTR and *OPRM1* A118G genotype at T1 between participating adolescents and drop-outs. Participants lost to follow-up were more likely to be male [χ^2^(1, *N* = 1399) = 4.28; *p* < .05], to be less educated [χ^2^(1, *N* = 1399) = 23.36; *p* < .001] and to be smokers [χ^2^(1, *N* = 1399) = 14.96; *p* < .001].

### Descriptive statistics

The means and standard deviations for the five measures of cue-induced craving and cognitive craving are presented in Table 2. The mean levels of both types of craving are relatively low (<1.6 on a 5-point scale), indicating that a large part of the sample reported little craving. On average, participants scored significantly higher on cognitive craving compared to cue-induced craving at all time points. The mean levels of both types of craving seem to increase over time. The percentage of regular smokers (those who smoked at least once a week) also increases over time, with 5.9 % at T1, 7.4 % at T2, 10.9 % at T3, 14.1 % at T4 and 15.9 % at T5. Descriptive findings for the genetic polymorphisms are also found in Table 2.

**Table 2:** Descriptive statistics for dependent model variables (*N* = 396)

	T1	T2	T3	T4	T5
Cue-induced craving					
Mean	1.27	1.29	1.37	1.50	1.36
(SD)	(.59)	(.62)	(.73)	(.91)	(.70)
Skewness	3.37	2.94	2.63	2.01	2.43
(SD)	(.19)	(.18)	(.17)	(.16)	(.16)
Cognitive craving					
Mean	1.38	1.38	1.44	1.57	1.50
(SD)	(.70)	(.81)	(.81)	(.95)	(.85)
Skewness	2.67	2.85	2.39	1.81	1.88
(SD)	(.19)	(.18)	(.17)	(.17)	(.17)

For both measures: min = 1, max = 5

### Correlations

Correlations between the model variables are presented in Table 3. These findings show that the measures of cue-induced and cognitive craving show mostly significant positive correlations, with the exceptions of cue-induced craving at T2 and cognitive craving at T5 and of cue-induced craving at T5 and cognitive craving at T1. Regarding the relation between cue-induced and cognitive craving and the genotypes *DRD2* Taq1A, *DRD4* 48 bp VNTR and *OPRM1* A118G, significant correlations were found only for *DRD2* Taq1A. *DRD2* Taq1A was positively associated with cue-induced craving at T2 and T3 and with cognitive craving at T1 and T2, indicating that adolescent *DRD2* Taq1A A1-allele carriers report more craving. Parental smoking exposure at T1 was positively correlated with all craving measures, with the exception of cue-induced craving at T4 and T5 and cognitive craving at T5.

**Table 3:** Spearman and Pearson correlations between the model variables

	1.	2.	3.	4.	5.	6.	7.	8.	9.	10.	11.	12.	13.	14.	15.	16.	17.
1. Sex	-																
2. Age	-.04	-															
3. Education	-.09	*-.16*	-														
4. Ethnicity	-.06	-.08	*-.17*	-													
5. *DRD2* Taq1A	*-.10*	.10	-.05	-.06	-												
6. *DRD4* VNTR	.13	-.02	-.03	.04	-.04	-											
7. *OPRM1* A118G	.09	.00	.06	-.05	-.02	.06	-										
8. Parental exposure	.08	.05	-.12	-.01	-.04	.03	.02	-									
9. Cue Craving T1	.11	.04	-.04	.02	.13	.04	.08	*.19*	-								
10. Cue Craving T2	.15	*.15*	.01	-.08	*.15*	.07	.12	*.19*	**.53**	-							
11. Cue Craving T3	**.24**	.08	-.01	-.05	*.15*	-.02	-.01	*.20*	**.50**	**.67**	-						
12. Cue Craving T4	**.20**	.13	.02	-.06	.02	-.09	.09	.11	*.29*	**.53**	**.72**	-					
13. Cue Craving T5	*.14*	.03	-.04	.04	-.06	-.05	-.01	.13	*.29*	*.26*	**.35**	**.58**	-				
14. Cog Craving T1	.13	.09	-.07	-.02	*.18*	.03	.08	*.25*	**.82**	**.62**	**.58**	**.33**	.19	-			
15. Cog Craving T2	.08	.12	.02	-.03	*.19*	.06	.12	*.20*	**.42**	**.91**	**.72**	**.53**	**.25**	**.57**	-		
16. Cog Craving T3	*.16*	.04	-.02	.02	.11	-.01	.00	*.15*	**.43**	**.59**	**.89**	**.74**	**.32**	**.54**	**.67**	-	
17. Cog Craving T4	**.19**	.13	-.03	-.07	.06	-.12	.08	*.15*	**.35**	**.56**	**.68**	**.93**	**.55**	**.46**	**.59**	**.74**	-
18. Cog Craving T5	.10	.00	.01	.03	-.04	.01	.05	.11	**.37**	.19	**.43**	**.59**	**.77**	**.36**	*.22*	**.51**	**.65**

‘Cue’ refers to Cue-induced and ‘Cog’ refers to Cognitive; Correlations with *p* < .05 are in italics, correlations with *p* < .01 are in boldface. Spearman correlations are underlined, all other correlations are Pearson correlations.

### Model findings

First, we tested the latent growth model for cue-induced craving without predictors. The model showed a reasonable fit to the data (χ^2^ [df = 10, *p* < .001] = 144.37, CFI = .87, RMSEA = .07). The means and variances of both intercept and slope were significant (respectively, mean = 1.21, *p* < .001; variance = .17, *p* < .01 and mean = .08, *p* < .001; variance = .06, *p* < .001), suggesting that the participants scored greater than zero on craving symptoms at baseline, that craving generally increased over time, and that participants differed around the means. The association between the intercept and the slope was not significant (β = −.20, *p* = .20). Quadratic trends were also examined but were not found to be significant.

The model for cognitive craving showed a good fit to the data (χ^2^ [df = 10, *p* < .001] = 155.58, CFI = .94, RMSEA = .04). The means and variances of both intercept and slope were significant (respectively, mean = 1.31, *p* < .001; variance = .28, *p* < .01 and mean = .06, *p* < .01; variance = .06, *p* < .001), suggesting that the participants scored greater than zero on craving symptoms at baseline, that craving generally increased over time, and that participants differ around the means. The association between the intercept and the slope was not significant (β = −.17, *p* < .39). Finally, the possibility of a quadratic trend was examined, but this was not found to be significant.

Second, the predictors and control variables were added to the growth models of cue-induced and cognitive craving. These models fit the data well (χ^2^ [df = 36, *p* = .27] = 40.64, CFI = .98, RMSEA = .02; χ^2^ [df = 36, *p* = .29] = 40.15, CFI = .98, RMSEA = .02). The control variable of smoking behavior, as measured at T1, was positively associated with the initial values of both cue-induced and cognitive craving (see Table 4). *DRD2* Taq1A was marginally associated (*p* = .01) with the intercept of cognitive craving. *DRD2* Taq1A A1-carriers seem to have higher initial cognitive craving levels. No other associations between the parental smoke exposure, *DRD2* Taq1A, *DRD4* 48 bp VNTR, *OPRM1* A118G and control variables on the one hand, and the slopes of cue-induced and cognitive craving on the other hand, were found.

**Table 4:** Standardized estimates for control variables genotypes and parental smoking exposure on the intercepts and slopes of cue-induced craving and cognitive craving

	Intercept cue-induced craving	Linear slope cue-induced craving	Intercept cognitive craving	Linear slope cognitive craving
Step 1				
Sex	.05	.19	.10	.10
Education	-.05	.10	.03	.06
Ethnicity	.05	-.08	.07	-.11
Age of smoking initiation	-.13	-.19	-.08	-.18
Baseline smoking	.81**	-.31	.72**	-.35
*DRD2* Taq1A	.15	-.11	.22*	-.21
*DRD4* 48 bp VNTR	.06	-.06	.09	-.14
*OPRM1* A118G	.08	.01	.09	.02
Parental smoke exposure	.07	.08	.12	-.01
Explained variance (R2)	76 %	21 %	65 %	27 %
Step 2				
DRD2**Parental smoke exposure	.55	-.76**	.52	-.96**
DRD4**Parental smoke exposure	.32	.25	.49	-.08
OPRM1**Parental smoke exposure	.02	-.30	-.20	-.18

Gender: 0 = male and 1 = female, Ethnicity: 0 = Dutch and 1 = Not Dutch

*p = .01, **p < .001

**Fig. 1:**
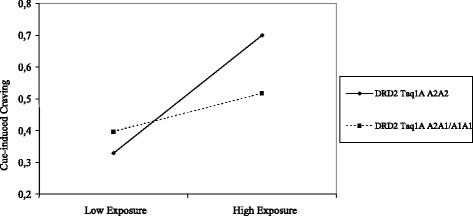
The moderating effect of DRD2 Taq1A and parental smoke exposure on the slope of cue-induced craving

**Fig. 2:**
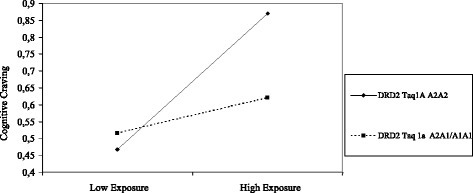
The moderating effect of DRD2 Taq1A and parental smoke exposure on the slope of cognitive craving

In a final step, the interactions were included in the model. For both cue-induced and cognitive craving, significant interaction effects were found for *DRD2* Taq1A with parental smoke exposure (Table 4). Adolescent A1-allele carriers do not seem to be influenced by the environment with regard to the development of cue-induced and cognitive craving (Figs. 1 and 2). Adolescents with the A2A2 genotype who are exposed to parental smoking increase most profoundly in craving over time. No significant interaction effects were found between *DRD4* 48 bp VNTR and parental smoke exposure on either craving measure. Similarly, no significant interaction effects were found for *OPRM1* A118G and parental smoke exposure on either of the measures.

## Discussion

As expected, we found that early adolescents’ cognitive as well as cue-induced craving symptoms increased over time. The occurrence and increase of craving symptoms conforms to the notion of the SHM that craving is commonly the first symptom of dependence among novice smokers [7]. Thus, urges to smoke are to be expected even in irregular smokers. This is conceivable since low-frequency smokers might not smoke at all if they did not experience cravings [65]. Even though both cue-induced and cognitive craving levels were relatively low, our findings indicate that these craving symptoms may already be present in early adolescent novice smokers and significantly increase within a short period of time.

No significant main effects of the *DRD2* Taq1A, *DRD4* 48 bp VNTR and *OPRM1* A118G genotypes were found on the baseline levels or on changes in craving. The absence of these main gene effects is in line with previous studies that suggested that genetic predispositions are most likely to increase the development of dependence if specific environmental factors are present [43, 44]. In our study, we indeed found evidence of an interaction between the *DRD2* Taq1A polymorphism and parental smoke exposure in the development of cognitive and cue-induced craving symptoms over time. The absence of a direct effect of parental smoke exposure on craving, however, was contrary to our expectations, since previous studies found that environmental smoking increases the risk for both smoking initiation and nicotine dependence in the future [40, 66–68]. However, adolescents who were homozygous for the *DRD2* Taq1A A2-allele and were also highly exposed to parental smoking did show a stronger increase of both cognitive craving and cue-induced craving over time. Although *DRD2* Taq1A interactions with environmental factors have rarely been studied in regard to smoking and nicotine dependence, our findings are in line with studies showing *DRD2* Taq1A to interact with environmental factors in shaping addictive behaviors or personality traits related to addictive behaviors. For example, interactions between *DRD2* Taq1A and both pre- and postnatal tobacco smoke exposure were found in the development of attention, irritable reactivity and self-regulation [69]. Also, *DRD2* Taq1A was found to interact with parental rule-setting in shaping adolescents’ alcohol use [70].

With regard to *DRD2* Taq1A, we found that in the case of no or low parental smoke exposure, both A1-allele carriers and those who were homozygous for the A2-allele show relatively little increase in craving over time. However, when exposure to parental smoking is high, those with homozygous A2-alleles show a significantly larger increase in both cognitive and cue-induced craving over time compared to A1-allele carriers. This finding is remarkable, since previous studies showed that individuals with the A1-allele have reduced brain *DRD2* Taq1A availability and less dopaminergic activity compared to those who are homozygous for the A2-allele [71]. It is possible that A1-carriers may compensate for this reduced sense of reward by using substances; in other words, they experience more rewarding feelings from the dopamine-enhancing effects of substance use. Adolescent smokers with the A1-allele indeed seem more likely to progress to smoking persistence and, eventually, dependence [13]. However, we found that, in the case of the development of initial craving symptoms, homozygosity for the *DRD2* Taq1A A2-allele is associated with a higher vulnerability to develop these symptoms over time if exposed to parental smoking. A1-carriers seem to develop craving symptoms largely independent of parental smoke exposure. Parental smoke exposure might thus be a catalyst of craving for adolescents homozygous for the *DRD2* Taq1A A2-allele, who are proposed to experience less rewarding feelings from nicotine and are thus less likely to develop dependence on their own (i.e., separate from environmental cues or influences).

No effect of *OPRM1* A118G on craving was found. These results are not in line with a recent systematic review on the *OPRM1* A118G polymorphism in nicotine addiction [20], which concluded that, although effects are generally small and mixed, the *OPRM1* A118G is associated with higher dopaminergic activity and feelings of reward in response to nicotine. Moreover, our findings do not corroborate the incentive salience theory [18], which suggests that ‘liking’ processes (allegedly associated with opioid neurotransmission) are especially relevant in the initiation phase of smoking behaviour. Even though craving is generally the first symptom of dependence, even earlier phenotypes of the risk of nicotine dependence can be distinguished, such as sensitivity to the first dose of nicotine (i.e., experience of rewarding or aversive sensations). Initial sensitivity to nicotine constitutes an early predictor of the vulnerability to developing nicotine dependence among novice smokers [72–75]. A recent study found that the *OPRM1* A118G genotype modulated initial responses to nicotine [76]. Carriers of the G-allele of the *OPRM1* A118G polymorphism were significantly more likely to report liking in response to initial smoking. It could be that the opioid system is mostly important with regard to an individual’s very first experience with nicotine, whereas the dopamine system becomes more prominent soon after initiation.

Also contrary to our expectations, no significant association was observed between the *DRD4* 48 bp VTNR polymorphism and the development of craving. *DRD2* Taq1A and *DRD4* 48 bp VNTR have been previously found to be differentially related to refined phenotypes in adolescent smoking [13]. In particular, 15-year olds with the > 7 repeats allele of the *DRD4* 48 bp VNTR were found to begin smoking at an earlier age and to have significantly higher rates of smoking initiation. Among adolescents who had ever smoked, the likelihood of smoking continuation and nicotine dependence was mostly found to be related to allelic variation in *DRD2* Taq1A. No studies so far have found a direct association between *DRD4* 48 bp VNTR and measures of nicotine dependence in adolescents. The variability in effects of *DRD2* Taq1A and *DRD4* 48 bp VNTR can be further substantiated by the finding that *DRD4* 48 bp VNTR is associated with a dopaminergically modulated and heritable tendency toward excitement in response to new experiences, which might explain the previously found associations with smoking initiation [14, 77]. Finally, even though an in vivo cue reactivity study among adult smokers found support for variability in the susceptibility to environmental smoking cues among different *DRD4* 48 bp VNTR genotypes in relation to craving among adult smokers [10], this effect was not replicated in a study focusing on reactivity to dynamic smoking cues in movies among college students [78]. The researchers use the heaviness of smoking to explain the lack of an interaction effect of cue-exposure and *DRD4* 48 bp VNTR. It is possible that an effect between smoke exposure and *DRD4* 48 bp VNTR can only be found among heavy smokers.

### Limitations

In this study, craving was measured by means of self-report. Although self-report measures are viewed as the most reliable and valid assessments of subjective craving, they are still subject to such limitations as individuals’ unwillingness or inability to report on internal processes [79, 80]. Second, the found gene–environment interaction might partly reflect a gene–environment correlation (rGE), since parents’ smoking behavior may reflect their genotype, which they may partly pass on to their offspring. Separation of these two types of gene–environment relationship has posed a major difficulty in quantitative genetic research. With molecular genetics, it is possible to infer rGE because it can be examined directly with respect to the identified G and the identified E [81]. We found no significant correlations between differences in *DRD2* Taq1A, *DRD4* 48 bp VNTR and *OPRM1* A118G genotypes of adolescents and their parental smoking exposure. Third, single nucleotide polymorphisms (SNPs) were tested, whereas multiple loci might be involved in the development of initial dependence symptoms. Because of linkage disequilibrium (i.e., non-random association between alleles), genotyping several SNPs within the *ANKK1* gene (where the polymorphism DRD2 Taq1 resides) and adjacent genes would be necessary to provide insight into other associated variants. Also, population stratification [82] cannot be completely ruled out. However, we do emphasize that population stratification is unlikely because only 4 % of the adolescents in this study were born outside the Netherlands. Finally, it should be noted that the present sample size was rather small, which may have resulted in an increased risk of type II error (i.e., false negatives). This may explain why no significant main effects of *DRD2* Taq1A, *DRD4* 48 bp VNTR or *OPRM1* A118G or significant interaction effects including these three polymorphisms have been found. Therefore, our findings need to be replicated in studies with larger sample sizes.

## Conclusions

In sum, parental smoking increases the chances of developing dependence more rapidly for young adolescents who are supposedly less sensitive to the rewarding effects of nicotine according to their *DRD2* Taq1A genotype. It is thus pivotal for these children not to be exposed to smoking.

## Additional file

Additional file 1:
**Questionnaire.** (DOC 176 kb)
