# Supplementary material for: Parental smoke exposure and the development of nicotine craving in adolescent novice smokers: the roles of DRD2, DRD4, and OPRM1 genotypes
Source: BMC Pulm Med. 2015 Oct 8;15:115. doi: 10.1186/s12890-015-0114-z (PMC4599744; doi:10.1186/s12890-015-0114-z)
Supplement: Additional file 1: — Questionnaire. (DOC 176 kb) [file 12890_2015_114_MOESM1_ESM.doc]

###
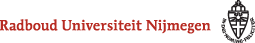


Questionnaire

Youth and Lifestyle

March 2011


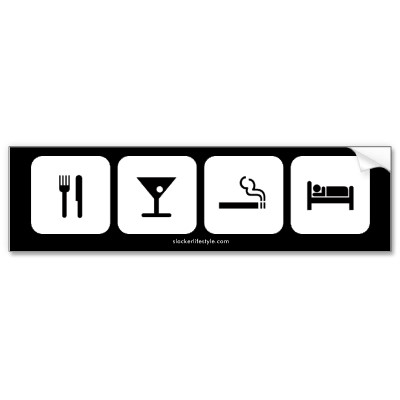


#### General Questions

|  |
| --- |
| Sex 🞏 boy 🞏 girl |
| Date of Birth: ……………………………………………………….. |

| **What is your current level of education?** | | |
| --- | --- | --- |
| 🞏 University preparatory |  Different, namely…………… |  |
| 🞏 Senior general |  |  |
| 🞏 Junior general |  |  |
| 🞏 Preparatory vocational |  |  |

| **In which country where you born?** |
| --- |
| 🞏 Netherlands |
| 🞏 Suriname |
| 🞏 Dutch Antillen or Aruba |
| 🞏 Morocco |
| 🞏 Turkye |
| 🞏 other country: _______________ |

| **In which country was your mother born?** |
| --- |
| 🞏 Netherlands |
| 🞏 Suriname |
| 🞏 Dutch Antillen or Aruba |
| 🞏 Morocco |
| 🞏 Turkye |
| 🞏 other country: _______________ |

| **In which country was your father born?** |
| --- |
| 🞏 Netherlands |
| 🞏 Suriname |
| 🞏 Dutch Antillen or Aruba |
| 🞏 Morocco |
| 🞏 Turkye |
| 🞏 other country: _______________ |

#### Smoking

**We’d like to ask you some questions about smoking. All questions are on the smoking of cigarettes**

| **1. Does your father smoke?** | | | |
| --- | --- | --- | --- |
| 🞏 No, my father never smoked 🡺*go to question 4b* | | | 🞏 Yes, 6-10 cigarettes per day |
| 🞏 No, my father has quit smoking 🡺*go to question 4a* | | | 🞏 Yes, 11-20 cigarettes per day |
| 🞏 Yes, but less than 1 cigarette per day | | | 🞏 Yes, 21-30 cigarettes per day |
| 🞏 Yes, 1-5 cigarettes per day | | | 🞏 Yes, > than 31 cigarettes per day |
|  | | |  |
|  |  | | |
| **2. Does your father smoke in your presence?** | | | |
| 🞏 No 🡺 *go to question 4b* | | 🞏 Yes | |
|  | |  | |
|  | |  | |
| **3. Where does your father smoke in your presence** *(more answers are possible)* | | | |
| 🞏 At home, in the kitchen/living room | | 🞏 At the home of friends or family | |
| 🞏 At home, in another space | | 🞏 In the street | |
| 🞏 At home, but outside | | 🞏 On parties | |
| 🞏 In the car | | 🞏 In café’s or restaurants | |
|  | |  | |
|  | |  | |
|  | |  | |
| **4a. How old were you when your father quit smoking?** | | | |
| I was ­­_______ years old | |  | |
| 🞏 That was before I was born | |  | |
|  | |  | |
| **4b. How much time do you spend with your father per day?** | | | |
| 🞏 Less than half an hour | | 🞏 About three hours | |
| 🞏 About half an hour | | 🞏 About 4 hours | |
| 🞏 About 1 hour | | 🞏 About 5 hours | |
| 🞏 About 2 hours | | 🞏 6 hours or more | |
|  | |  | |
|  | |  | |
| **5.**  **Does your mother smoke?** | | | |
|  No, my mother never smoked go to question 8b | | |  Yes, 6-10 cigarettes per day |
|  No, my mother has quit smoking go to question 8a | | |  Yes, 11-20 cigarettes per day |
|  Yes, but less than 1 cigarette per day | | |  Yes, 21-30 cigarettes per day |
|  Yes, 1-5 cigarettes per day | | |  Yes, > than 31 cigarettes per day |
|  | | |  |
|  | | |  |
| **6.**  **Does your mother smoke in your presence?** | | | |
| 🞏 No 🡺*Go to question 8b* | | 🞏 Yes | |
|  | |  | |
|  | |  | |
| **7.**  **Where does your mother smoke in your presence** *(more answers are possible)* | | | |
|  At home, in the kitchen/living room | |  At the home of friends or family | |
|  At home, in another space | |  In the street | |
|  At home, but outside | |  On parties | |
|  In the car | |  In café’s or restaurants | |
|  | |  | |

| **8a.**  **How old were you when your mother quit smoking?** | |
| --- | --- |
| I was ­­_______ years old |  |
| 🞏 That was before I was born |  |
|  |  |

| **8b.**  **How much time do you spend with your mother per day?** | |
| --- | --- |
|  Less than half an hour |  About three hours |
|  About half an hour |  About 4 hours |
|  About 1 hour |  About 5 hours |
|  About 2 hours |  6 hours or more |
|  |  |
|  |  |

| **9. Which of the following statements best describes you? (you can only pick one answer)** |
| --- |
| 🞏 I smoke at least once a day |
| 🞏 I don’t smoke daily, but at least once per week |
| 🞏 I don’t smoke weekly, but at least once per month |
| 🞏 I smoke less than once per month |
| 🞏 I occasionally try out smoking |
| 🞏 I quit smoking after having smoked at least once per week |
| 🞏 I quit smoking, I always smoked less than once per week |
| 🞏 I have tried smoking once or twice, but I no longer smoke |
| 🞏 I never smoked, not even one puff 🡺 *You have now finished the questionnaire* |

| **10. At what age did you smoke for the first time, if only one puff?** | ..............…………years old |
| --- | --- |

| **10a How long ago did you smoke for the first time?** |
| --- |
| 🞏 Less than 1 month ago  🞏 About 1 month ago  🞏 About 2 months ago  🞏 About 3 months ago  🞏 About 4 months ago  🞏 Longer than 4 months ago |

| **11. How many cigarettes do you smoke on average per week?** | ………cigarettes |
| --- | --- |

If you only smoke(d) occasionally, for example only during specific occasions, the following questions may seem to apply less to you. However, we ask you to try to answer the questions as well as possible.

| 12. Do you inhale?? | | | |
| --- | --- | --- | --- |
| 🞏 Never | 🞏 Seldom | 🞏 Often | 🞏 Always |

| 13. How soon after you wake up do you smoke your first cigarette? | |
| --- | --- |
| 🞏 Within 5 minutes | 🞏 Within 31-60 minutes |
| 🞏 Within 6-30 minutes | 🞏 After 60 minutes |

| 14. Which cigarette would you hate to give up? | |
| --- | --- |
| 🞏 First cigarette in the morning | 🞏 Another cigarette |

| **15. How many cigarettes a day do you smoke??** | |
| --- | --- |
| 🞏 Less than 1 cigarette per day | 🞏 11-20 cigarettes per day |
| 🞏 1-5 cigarettes per day | 🞏 21-30 cigarettes per day |
| 🞏 6-10 cigarettes per day | 🞏 31 or more cigarettes per day |

| 16. Do you smoke more during the first 2 hours than during the rest of the day? | |
| --- | --- |
| 🞏 No | 🞏 Yes |

| 17. Do you smoke if you are so ill that you are in bed most of the day? | |
| --- | --- |
| 🞏 No | 🞏 Yes |

| 18. Have you ever felt like you were addicted to tobacco? | | | |
| --- | --- | --- | --- |
| 🞏 Never | 🞏 Seldom | 🞏 Often | 🞏 Always |

| 19. Do you ever have strong cravings to smoke? | | | |
| --- | --- | --- | --- |
| 🞏 Never | 🞏 Seldom | 🞏 Often | 🞏 Always |

| 20. Have you ever felt like you really needed a cigarette? | | | |
| --- | --- | --- | --- |
| 🞏 Never | 🞏 Seldom | 🞏 Often | 🞏 Always |

| 21. Do you smoke now because it is really hard to quit? | | | |
| --- | --- | --- | --- |
| 🞏 Not at all | 🞏 A little | 🞏 Somewhat | 🞏 Very |
|  |  |  |  |
| 22. Do you find it difficult to refrain from smoking in places where it is forbidden (church, library, movies, school etc.)? | | | |
| 🞏 Not at all | 🞏 A little | 🞏 Somewhat | 🞏 Very |

| **23. When you haven’t used tobacco for a while … OR**  **When you tried to stop smoking** | **Never** | **Seldom** | **Often** | **Always** |
| --- | --- | --- | --- | --- |
| Did you find it hard to concentrate because you couldn't smoke? | 🞏 | 🞏 | 🞏 | 🞏 |
| Did you feel more irritable because you couldn't smoke? | 🞏 | 🞏 | 🞏 | 🞏 |
| Did you feel a strong need or urge to smoke? | 🞏 | 🞏 | 🞏 | 🞏 |
| Did you feel nervous, restless or anxious because you couldn't  smoke? | 🞏 | 🞏 | 🞏 | 🞏 |

| **35. How often do you think or experience the following?** | **Never** | **Some**  **times** | **Regu**  **larly** | Often | **Very often** |
| --- | --- | --- | --- | --- | --- |
| I desire to smoke a cigarette | 🞏 | 🞏 | 🞏 | 🞏 | 🞏 |
| I think about the nice feeling of deeply inhaling the smoke from a cigarette | 🞏 | 🞏 | 🞏 | 🞏 | 🞏 |
| I miss a cigarette | 🞏 | 🞏 | 🞏 | 🞏 | 🞏 |
| I look forward to lighting a cigarette | 🞏 | 🞏 | 🞏 | 🞏 | 🞏 |
| I desire to inhale the smoke from a cigarette | 🞏 | 🞏 | 🞏 | 🞏 | 🞏 |

| **36. How often do you think or experience the following?** | **Never** | **Some**  **times** | **Regu**  **larly** | Often | **Very often** |
| --- | --- | --- | --- | --- | --- |
| When I feel stressed I want a cigarette | 🞏 | 🞏 | 🞏 | 🞏 | 🞏 |
| When I see other people smoking I want a cigarette | 🞏 | 🞏 | 🞏 | 🞏 | 🞏 |
| When I smell cigarette smoke I want a cigarette | 🞏 | 🞏 | 🞏 | 🞏 | 🞏 |
| After eating I want a cigarette | 🞏 | 🞏 | 🞏 | 🞏 | 🞏 |
